# 3D ^31^P MR spectroscopic imaging of the human brain at 3 T with a ^31^P receive array: An assessment of ^1^H decoupling, T_1_ relaxation times, ^1^H‐^31^P nuclear Overhauser effects and NAD^+^

**DOI:** 10.1002/nbm.4169

**Published:** 2019-09-13

**Authors:** Tom H. Peeters, Mark J. van Uden, Anne Rijpma, Tom W.J. Scheenen, Arend Heerschap

**Affiliations:** ^1^ Department of Radiology and Nuclear Medicine Radboud University Medical Center Nijmegen The Netherlands; ^2^ Department of Geriatric Medicine Radboud University Medical Center Nijmegen The Netherlands; ^3^ Radboudumc Alzheimer Center, Donders Institute for Brain, Cognition and Behaviour Radboud University Medical Center Nijmegen The Netherlands; ^4^ Erwin L. Hahn Institute University Hospital Duisburg‐Essen Essen Germany

**Keywords:** brain, ^1^H decoupling, nuclear Overhauser effect, ^31^P MR spectroscopic imaging, T_1_, 3 T

## Abstract

^31^P MR spectroscopic imaging (MRSI) is a versatile technique to study phospholipid precursors and energy metabolism in the healthy and diseased human brain. However, mainly due to its low sensitivity, ^31^P MRSI is currently limited to research purposes. To obtain 3D ^31^P MRSI spectra with improved signal‐to‐noise ratio on clinical 3 T MR systems, we used a coil combination consisting of a dual‐tuned birdcage transmit coil and a ^31^P eight‐channel phased‐array receive insert. To further increase resolution and sensitivity we applied WALTZ4 ^1^H decoupling and continuous wave nuclear Overhauser effect (NOE) enhancement and acquired high‐quality MRSI spectra with nominal voxel volumes of ~ 17.6 cm^3^ (effective voxel volume ~ 51 cm^3^) in a clinically relevant measurement time of ~ 13 minutes, without exceeding SAR limits. Steady‐state NOE enhancements ranged from 15 ± 9% (γ‐ATP) and 33 ± 3% (phosphocreatine) to 48 ± 11% (phosphoethanolamine). Because of these improvements, we resolved and detected all ^31^P signals of metabolites that have also been reported for ultrahigh field strengths, including resonances for NAD^+^, NADH and extracellular inorganic phosphate. T_1_ times of extracellular inorganic phosphate were longer than for intracellular inorganic phosphate (3.8 ± 1.4s vs 1.8 ± 0.65 seconds). A comparison of measured T_1_ relaxation times and NOE enhancements at 3 T with published values between 1.5 and 9.4 T indicates that T_1_ relaxation of ^31^P metabolite spins in the human brain is dominated by dipolar relaxation for this field strength range. Even although intrinsic sensitivity is higher at ultrahigh fields, we demonstrate that at a clinical field strength of 3 T, similar ^31^P MRSI information content can be obtained using a sophisticated coil design combined with ^1^H decoupling and NOE enhancement.

## Graphical abstract

We performed 3D ^31^P MRSI of the human brain at 3 T employing a ^1^H/^31^P dual‐tuned birdcage transmit coil and a ^31^P 8‐channel phased‐array receive insert to acquire high‐quality ^31^P MR spectra with and without ^1^H‐decoupling and/or ^1^H‐^31^P NOE. The well‐resolved spectra allowed to analyze two peaks for inorganic phosphate and the NAD^+^/NADH redox ratio. Localized T_1_ relaxation times and NOE enhancements of ^31^P spins were measured and compared with those acquired at other field strengths, indicating little effect of chemical shift anisotropy relaxation.

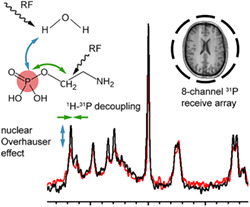

**Abbreviations used**
ATP: adenosine triphosphate
CSA: chemical shift anisotropy
CW: continuous wave
FID: free induction decay
FOV: field of view
GPC: glycerophosphocholine
GPE: glycerophosphoethanolamine
imP: immobile phosphates
MRSI: magnetic resonance spectroscopic imaging
NAD: nicotinamide adenine dinucleotide
NADH: NAD reduced
NAD+: NAD oxidized
NOE: nuclear Overhauser effect
OTP: occipito–temporal–parietal
PC: phosphocholine
PCr: phosphocreatine
PDE: phosphodiesters
PE: phosphoethanolamine
Pi: inorganic phosphate
Pi_ex_: extracellular inorganic phosphate
Pi_in_: intracellular inorganic phosphate
PME, phosphomonoesters
SAR: specific absorption rate
SD: standard deviation
SEM: standard error of the mean
SNR: signal‐to‐noise ratio
TR: repetition time
UDPG: uridine diphosphate glucose

## 1 INTRODUCTION

In vivo phosphorus magnetic resonance spectroscopy (^31^P MRS) of living tissues enables monitoring of phosphorus‐containing compounds involved in membrane synthesis and energy metabolism. In addition, from the chemical shift of the ^31^P resonances of some of these compounds, intracellular pH and magnesium content can be determined.^1^ Recently, it was also shown that at a magnetic field of 4 T or higher the cellular redox state of the brain can be determined from the nicotinamide dinucleotide (NAD(H)) resonances.^2^ ^31^P MRS of the human brain can provide unique information about various neurological diseases (eg,^3^, ^4^, ^5^, ^6^, ^7^, ^8^). However, because of a lower sensitivity and the need for additional hardware, ^31^P MRS is less frequently used than ^1^H MRS.^9^

Localized ^31^P MRS of the brain is commonly performed with surface or birdcage coils, integrated with ^1^H coils for MRI and shimming, and pulse sequences to select a single voxel or multiple voxels.^10^, ^11^, ^12^ To make up for the lower sensitivity of ^31^P compared with ^1^H MRS, larger voxels and/or longer acquisition times are usually employed. The intrinsic signal‐to‐noise ratio (SNR) can be improved by performing ^31^P MRS at a high main magnetic field and by using dedicated receive coils. Most clinical sites do not have access to expensive ultrahigh field MR systems (≥ 7 T) and therefore rely on adequate coil designs to optimize sensitivity. A phased array of receive coils encompassing the whole brain combined with a homogeneous ^31^P volume coil for transmit can boost SNR, in particular in superficial brain areas, while maintaining a large field of view (FOV).^10^, ^13^, ^14^ Such coil setups do not require high power adiabatic pulses for a homogeneous ^31^P excitation profile, but can be employed with rectangular pulses with low radiofrequency (RF) power instead. As a result, RF power deposition remains well below maximum specific absorption rates (SAR), which creates room for further spectral and sensitivity improvement by ^1^H irradiation techniques like ^1^H‐^31^P heteronuclear decoupling and nuclear Overhauser effect (NOE) enhancement.

Broadband ^1^H decoupling can improve spectral resolution and sensitivity, in particular at field strengths of 3 T or below for which the attainable ^31^P resonance linewidths of metabolites are of the same order as the ^1^H‐^31^P J‐coupling constants.^15^ This J‐coupling results in splitting of ^31^P resonances, which appears as a line broadening in in vivo ^31^P MR spectra and decreases spectral resolution. In ^1^H‐decoupled spectra, the ^1^H‐^31^P J‐coupling is eliminated by saturating the coupled proton spins with high‐power broadband ^1^H irradiation pulses during ^31^P signal acquisition. In this way, peak splitting is removed and spectral overlap is minimized, resulting in increased SNR and improved fitting accuracy.

NOE enhancement is achieved by the saturation of proton spins near ^31^P nuclei by applying low‐power proton irradiation at the water frequency prior to ^31^P signal acquisition. As a result of cross‐relaxation, polarization will be transferred from the saturated protons to the ^31^P nuclei of metabolites, which leads to an increase of the ^31^P signal.^16^

In this study we evaluated the benefits of using a volume transmit and phased array receive setup at 3 T to perform ^31^P MRSI with ^1^H decoupling and ^1^H‐^31^P NOE. As there are only limited data available on the effect of ^1^H decoupling on signal linewidth, on signal enhancement due to ^1^H‐^31^P NOE, and on T_1_ relaxation times of ^31^P spins in the brain at 3 T, we performed spatially resolved 3D ^31^P MRSI to determine the value of these variables for the resonances of all phosphorylated metabolites in the healthy human brain that can be detected. In addition, we investigated if we could determine the redox state of the brain from the ^31^P resonances of the oxidized and reduced form of nicotinamide adenine dinucleotide (NAD^+^ and NADH, respectively) at 3 T. Finally, we compared our results with those reported for other field strengths and discuss to what extent our ^31^P MRSI methodology at 3 T can compete with reported ^31^P MRSI at ultrahigh fields.

## 2 EXPERIMENTAL

In total, 12 volunteers participated in this study, which was conducted with the approval of the institutional review board of Radboud University Medical Center, Nijmegen. Age and gender of the volunteers are reported below.

### 2.1 Data acquisition

^31^P MRS experiments were performed on a Magnetom Prisma Fit 3 T MR system (Siemens Healthcare, Erlangen, Germany) with a customized coil setup as previously described.^14^ Briefly, this setup consists of an in‐house‐built eight‐channel ^31^P receive head‐array coil (inner diameter 23 cm, element size 10 x 20 cm) combined with a commercially available quadrature Tx/Rx ^1^H/^31^P birdcage coil (RAPID Biomedical, Würzburg, Germany). With this setup the ^1^H‐^31^P volume coil generates a homogeneous transmit field while ^31^P signals close to the elements are received with a high sensitivity by the eight‐channel array insert. In order to allow active detuning at the ^31^P frequency in both the transmit and receive coils, small adjustments were made to the commercial birdcage coil by the manufacturer. ^31^P transmit pulse calibration was performed by means of maximizing PCr signal intensity in a series of slice‐selective pulse‐acquire experiments with an incremental flip angle (TR = 15 seconds) covering the brain cortex.

Whole brain 3D ^31^P MRSI FID datasets were acquired with and without NOE or ^1^H decoupling pulses in six volunteers (male/female: 2/4, mean age: 31.3 ± 7.3 years). TR was set to 2000 ms. ^31^P spins were excited by a rectangular‐shaped excitation pulse (duration: 500 μs) with a flip angle of 40°. Dead time between the end of excitation and the start of FID acquisition was 100 μs, accommodating phase‐encoding gradients in three dimensions. FIDs (1024 data points, 512 ms) in a 10 x 10 x 10 matrix were averaged four times using Hamming‐weighted k‐space sampling. The FOV was centered on the brain midline and aligned parallel to the tangent to the anterior and posterior commissure. FOV dimensions were 260 x 260 x 260 mm^3^, resulting in nominal voxels of 17.6 cm^3^. This corresponds to an effective spherical voxel size of 51 cm^3^, which is defined as 64% of the point spread function area.^17^ The total measurement time was 13 minutes 8 seconds per dataset.

For NOE and ^1^H decoupling the proton frequency was centered on the water resonance. For steady‐state ^1^H‐^31^P NOE experiments, continuous wave (CW) irradiation was applied quasicontinuously (30 pulses, pulse duration 47.7 ms, interpulse delay 1 ms) prior to each ^31^P excitation pulse, with a duration of 1440 ms and a γB_1_ of 35 Hz. Proton decoupling was applied during the first 256 ms (50%) of the acquisition window and was achieved with a WALTZ4 decoupling scheme (γB_1_ of 250 Hz, decoupling bandwidth of 625 Hz).

T_1_ relaxation times of all observable ^31^P metabolites were assessed with a 2D MRSI slice‐selective FID progressive saturation experiment in six volunteers (male/female: 3/3, mean age: 32.0 ± 5.4 years). The pulse TR was changed in each experiment according to the following scheme: 550, 1000, 18 000, 9000, 2000, 5000 ms, with a fixed 90° flip angle. The acquisition matrix contained 8 x 8 voxels with a nominal size of 37.5 x 37.5 x 70 mm^3^. For better SNRs, experiments with a TR of 550 and 1000 ms were averaged 32 and 16 times, respectively; all of the other experiments consisted of eight averages.

### 2.2 Data postprocessing

FIDs of each receive element were combined in the time domain using the Brown combination.^18^ 3D ^31^P MRSI data were Hamming‐filtered and zerofilled to a 16 x 16 x 16 matrix before Fourier transformation. All data were evaluated with the software package syngo.via (Siemens Healthcare) and included postprocessing (zerofilling, phase correction, baseline correction and filtering [159 ms exponential filter]) and automatic peak fitting in the time domain. Prior knowledge for fitting of ^1^H‐decoupled and nondecoupled ^31^P MR spectra included the chemical shifts, relative peak heights of multiplets and ^31^P‐^31^P J‐coupling constants of the well‐resolved resonances of 12 metabolites, as described elsewhere.^1^, ^19^ The chemical shifts of the NADH and NAD^+^ resonances were fixed and related to the α‐ATP chemical shift. For NAD(H), linewidths were used as fitted for α‐ATP. Immobile phosphates (imP) were fitted with an additional peak at 2.25 ppm. In order to compare metabolite linewidths at half height, all ^31^P resonances in nonfiltered spectra—acquired with and without ^1^H decoupling—were fitted as singlets with a Gaussian shape.

Signal enhancement by the steady‐state ^1^H‐^31^P NOE was evaluated per volunteer in 20 different voxels of two transversal partitions of the 3D dataset covering the occipito–temporal–parietal (OTP) cortex of the brain. Per metabolite the enhancement was calculated as the percentage increase of the peak integral obtained with and without NOE or ^1^H decoupling:

$$\eta =\frac{S_{\text{enhanced}}-S_{\text{native}}}{S_{\text{native}}}\;\cdot 100\%$$

Averaged voxel values per volunteer were combined to obtain a mean enhancement (± standard deviation [SD]) for all six volunteers. In addition, average linewidths of all metabolite signals and SNR of PCr and α‐ATP were calculated from the same voxels. Linewidth was defined as the full width at half signal maximum. SNR was determined as the peak height divided by the SD of the noise.

Tissue pH was calculated from the difference in chemical shift (*δ*_*ppm*_) between the PCr and Pi resonance, according to:

$$\mathrm{pH}=pK_{a}+\mathrm{Log}\;\left(\frac{\left(\delta_{\mathrm{ppm}}-\delta_{a}\right)}{\left(\delta_{b}-\delta_{\mathrm{ppm}}\right)}\right)$$

with phosphoric acid dissociation constant *pK*_*a*_ = 6.73, and ^31^P limiting shifts *δ*_*a*_ = 3.275 and *δ*_*b*_ = 5.685 ppm.^20^ From all ^1^H‐decoupled datasets we selected 21 voxels to measure *δ*_*ppm*_ between the resonance of PCr and of both intracellular (Pi_in_) and extracellular (Pi_ex_) inorganic phosphate. Intra‐ and extracellular pH values were calculated and presented as mean values (± SD).

T_1_ relaxation times of all ^31^P metabolites were calculated in a large voxel centered in the occipital and parietal cortex. Signal integrals at different TRs were fitted with a nonlinear fitting routine in Matlab (The MathWorks Inc., Natick, MA, USA) to the monoexponential function

$$M\left(t\right)=M\left(0\right)\left(1-e^{\frac{-t}{T_{1}}}\right)$$

describing progressive saturation. *M*(*t*) is the signal intensity at time TR and *M*(0) at infinite TR. Fitted T_1_ values are reported only in cases where the goodness of fit (R^2^) was larger than 0.7.

## 3 RESULTS

### 3.1 Spectral resolution and ^1^H decoupling

**FIGURE 1:**
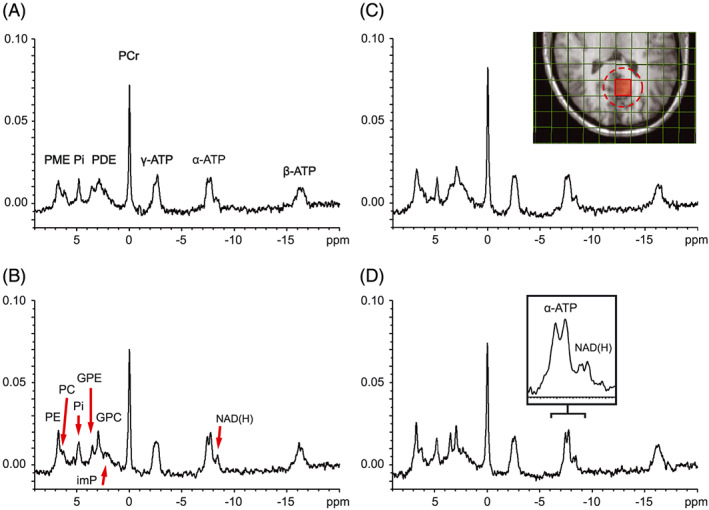
Human brain ^31^P MR spectra acquired with 3D MRSI with and without ^1^H irradiation. Line broadening is applied with a 2 Hz exponential filter. All spectra are from the nominal voxel shown in red in the inset of Figure 1C. The effective voxel size (~ 51 cc) is indicated with a dashed circle. (A) Spectrum obtained without ^1^H irradiation. (B) Spectrum obtained with WALTZ4 ^1^H decoupling during 50% of the acquisition time. The PME peak is better resolved in phosphoethanolamine (PE) and phosphocholine (PC) and the PDE peak in glycerophosphoethanolamine (GPE) and glycerophosphocholine (GPC). The individual resonances of α‐ATP and NAD(H) are also better resolved. (C) Spectrum obtained with ^1^H CW pulses applied during 1440 ms before the ^31^P excitation pulse. A clear NOE enhancement is observed for most ^31^P resonances. Compare with Figure 1A. (D) Spectrum obtained with a combination of ^1^H CW pulses 1440 ms prior to ^31^P excitation, and WALTZ4 ^1^H decoupling during 50% of the acquisition time. The inset is an enlargement of the resonance region of α‐ATP and NAD(H). imP, immobile phosphates; PME, phosphomonoesters; Pi, inorganic phosphate; PDE, phosphodiesters; PCr, phosphocreatine; NAD(H), nicotineamide adenine dinucleotide

In ^31^P MR spectra from voxels obtained of the human brain by 3D ^31^P MRSI at 3 T with an eight‐channel receive array, peaks can be observed for ATP, phosphocreatine (PCr), phosphodiesters (PDE), inorganic phosphate (Pi) and phosphomonoesters (PME) (Figure 1A). In these spectra, individual PDE and PME peaks are not well resolved due to ^1^H‐^31^P J‐coupling. By applying WALTZ4 ^1^H decoupling with the brain volume coil at the ^1^H frequency during the ^31^P acquisition time this coupling can be removed, eliminating the ^1^H splitting of the ^31^P peaks and resulting in higher peak intensities^15^ (Figure 1B). The ^1^H‐decoupled ^31^P spectra from MRSI voxels showed well‐resolved signals of phosphoethanolamine (PE), phosphocholine (PC), glycerophosphoethanolamine (GPE) and glycerophosphocholine (GPC) as well as a better resolved doublet for α‐ATP and for the multiplet consisting of the oxidized and reduced form of nicotinamide adenine dinucleotide (NAD^+^ and NADH, respectively). The ^1^H decoupling did not generate additional noise in the ^31^P spectra and could be performed within SAR limits at the applied experimental conditions.^14^

**FIGURE 2:**
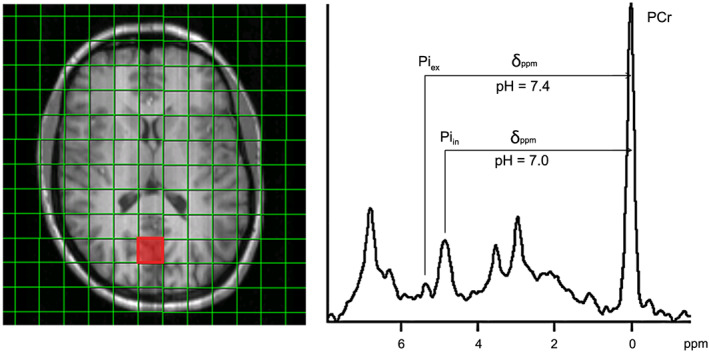
Inorganic phosphate resonances assigned to intracellular phosphate (Pi_in_) and extracellular phosphate (Pi_ex_) and the calculated corresponding pH values of these tissue compartments. The voxel location of the displayed ^1^H‐decoupled spectrum is indicated with a red square

For the ^1^H decoupled spectra of 20 voxels in the occipito‐temporal‐parietal (OTP) region of the brain (vide infra) of six volunteers we determined an average SNR for PCr of 51.4 ± 10.6 and for α‐ATP of 13.8 ± 1.8. Variations in SNR can be attributed to different distances between voxels and coil elements. The high SNR achieved with the receive array coil also facilitated the observation of a small phosphate peak resolved at about 0.4 to 0.6 ppm downfield from the intracellular Pi peak (Pi_in_) (Figure 2), which has been assigned to extracellular phosphate (Pi_ex_).^20^, ^22^ From the chemical shifts of Pi_ex_ and Pi_in_ in spectra of different voxels, we calculated a pH of between 7.3 and 7.5 and of ~ 7.0, respectively.

**FIGURE 3:**
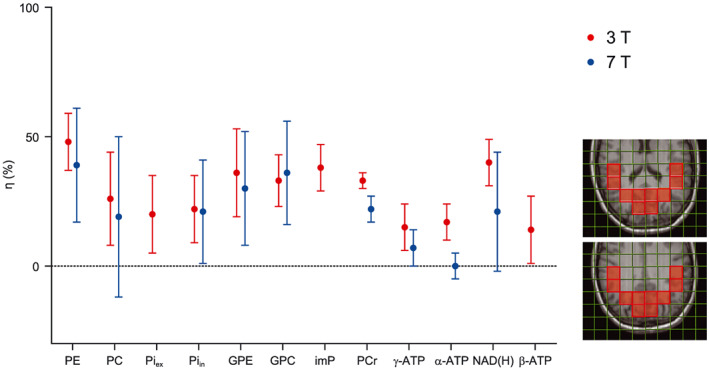
NOE enhancement factors of ^31^P metabolites at 3 T (red). For comparison, NOE enhancement factors at 7 T, reported by Lagemaat et al^21^ are displayed in blue. All evaluated voxels (OTP region) are projected on the T_1_‐weighted anatomical MR images (right)

As a result of ^1^H decoupling, the linewidths of peaks of phosphates that are J‐coupled with protons are decreased. In Table 1 we present an overview of the average fitted linewidths in native ^31^P MRSI data without ^1^H irradiation and in ^1^H‐decoupled spectra as determined for 20 nominal voxels in six volunteers (Figure 3). The average linewidth of PCr was 7.3 ± 2.9 Hz and did not change when ^1^H decoupling was applied, whereas the linewidths of the ^31^P resonances for PE, PC, GPE, GPC and α‐ATP, of which the phosphates are coupled to protons, decreased by 8.2 (PE and PC), 11.8 (GPE and GPC) and 4.9 Hz (α‐ATP).

**TABLE 1:** Average linewidths (± SD) of fitted metabolites in ^31^P MRSI spectra in the brain at 3 T (Hz) without and with ^1^H decoupling. Voxel locations of included spectra are indicated in Figure 3. The phosphocholine (PC) and phosphoethanolamine (PE) linewidths were linked in the prior knowledge set, and are therefore equal. The same holds for glycerophosphocholine (GPC) and glycerophosphoethanolamine (GPE)

	Native MRSI		^1^H‐decoupled	
	Linewidth (Hz)		Linewidth (Hz)	
PE and PC	22.8	(± 2.3)	14.6	(± 5.6)
Pi	15.5	(± 4.1)	15.5	(± 5.1)
GPE and GPC	22.6	(± 2.7)	10.8	(± 5.0)
imP	62.6	(± 17.6)	69.2	(± 12.3)
PCr	7.3	(± 2.9)	7.3	(± 2.8)
γ‐ATP	19.3	(± 1.6)	19.1	(± 2.5)
α‐ATP	17.2	(± 3.1)	12.3	(± 2.8)
β‐ATP	30.1	(± 7.0)	29.1	(± 4.1)

Abbreviations: ATP, adenosine triphosphate; imP, immobile phosphates; MRSI, magnetic resonance spectroscopic imaging; PCr, phosphocreatine; Pi, inorganic phosphate

### 3.2 Signal enhancement by ^1^H‐^31^P NOE

Next to the use of a ^31^P receive array, the SNR can also be enhanced by the ^1^H‐^31^P NOE. An example of an NOE‐enhanced spectrum is displayed in Figure 1C. We characterized typical NOE enhancement factors for all detectable metabolites at 3 T. When applying CW saturation at the water frequency for 1.5 seconds prior to the acquisition, we observed average ^1^H‐^31^P NOE enhancements ranging from 48 ± 11% for PE and 33 ± 3% for PCr to 15 ± 9% for γ‐ATP for selected voxels in the OTP region (Figure 3). The enhancement factor of total NAD(H) is reported, as the resonances of NAD^+^ and NADH are difficult to analyze separately without ^1^H decoupling. A complete overview of averaged values from six volunteers is presented in Table 2. A comparison of our NOE data with those previously obtained at 7 T shows that the enhancements are similar or slightly higher^21^ (Figure 3). As we averaged voxels with an overlapping effective voxel size, reported SDs at 3 T are relatively small.

**TABLE 2:** Overview of average steady‐state nuclear Overhauser effect (NOE) enhancements (% ± SD) in the occipito–temporal–parietal (OTP) region of the brain at 3 T

Metabolite	^1^H‐^31^P NOE enhancement (%)	
PE	48	(± 11)
PC	26	(± 18)
Pi_ex_	20	(± 15)
Pi_in_	22	(± 13)
GPE	36	(± 17)
GPC	33	(± 10)
imP	38	(± 9)
PCr	33	(± 3)
yATP	15	(± 9)
aATP	17	(± 7)
NAD(H)	40	(± 9)
bATP	14	(± 13)

Abbreviations: ATP, adenosine triphosphate; GPC, glycerophosphocholine; GPE, glycerophosphoethanolamine; imP, immobile phosphates; PC, phosphocholine; PCr, phosphocreatine; PE, phosphoethanolamine; Pi_ex_, extracellular inorganic phosphate; Pi_in_, intracellular inorganic phosphate; NAD(H), nicotinamide adenine dinucleotide (oxidised and reduced)

Because the combined coil setup allows application of rectangular ^31^P excitation pulses while preserving a homogeneous excitation profile, RF power deposition is low. Therefore, we were able to apply WALTZ4 ^1^H decoupling pulses in combination with CW ^1^H‐^31^P NOE pulses, without exceeding SAR limits at a TR of 2 seconds.^14^ An example of a ^1^H‐decoupled spectrum with NOE enhancement is shown in Figure 1D.

**FIGURE 4:**
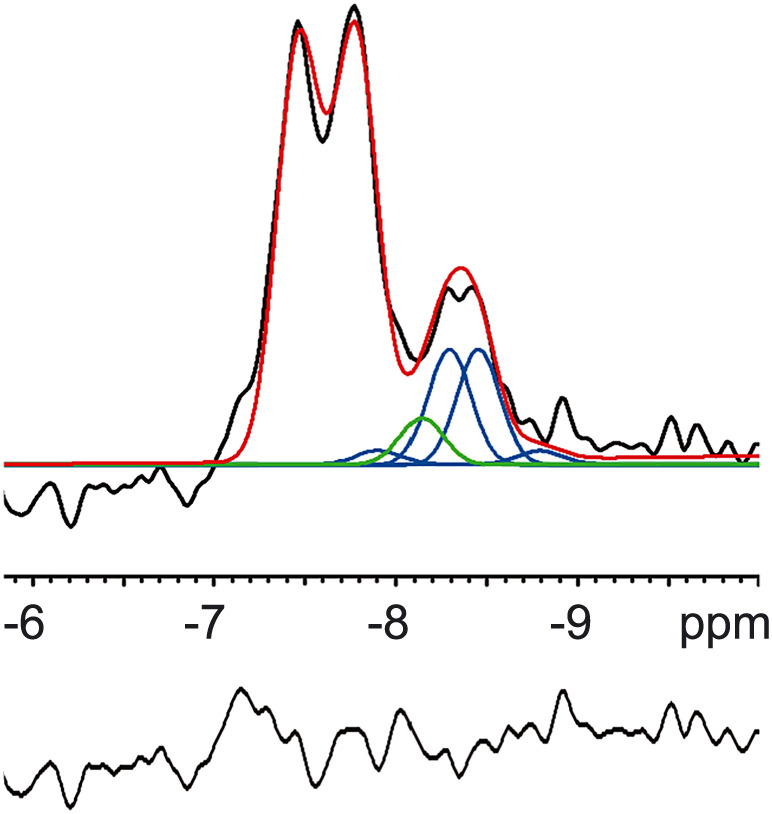
Example of the α‐ATP and NAD(H) region of an in vivo ^31^P MR spectrum obtained from a voxel in the occipital region of the human brain at 3 T. The spectrum (black) was filtered with a 2 Hz exponential filter. Relative peak heights of NAD^+^ were fixed according to.^19^ Chemical shift of the NAD(H) resonances were fixed with respect to the α‐ATP chemical shift. Linewidths of NAD(H) were equal to the linewidth of α‐ATP. The fitting result is shown in red. The resonances of α‐ATP, NAD^+^ (blue) and NADH (green) could be spectrally resolved when NOE‐enhancement and ^1^H‐decoupling were applied and prior knowledge of the NAD^+^ and NADH resonances was implemented in their fitting. The bottom line (black) shows the fitting residue

In ^1^H‐decoupled ^31^P spectra with NOE enhancement (*n* = 4), we were able to separately assess the resonances for NAD^+^ and NADH (Figure 4). From the integral of these metabolite resonances we determined an average cellular redox state NAD^+^/NADH of 5.7 ± 0.9 for the selected voxels indicated in Figure 3. We also calculated the tissue concentrations of both metabolites using α‐ATP = 3.0 mM as an internal reference,^22^ and taking into account the number of contributing phosphate groups (1:2) as well as the determined NOE enhancements of α‐ATP (17%) and NAD(H) (40%). We found tissue concentrations of 0.41 ± 0.03 and 0.07 ± 0.01 mM for NAD^+^ and NADH, respectively.

Proton irradiation by ^1^H decoupling also saturates proton spins of nearby ^31^P nuclei. At short TRs, this can also induce NOE enhancement.^15^, ^23^ For PCr, a resonance that is not influenced in shape by decoupling, we observed a NOE enhancement of 17 ± 4% due to decoupling at a TR of 2 seconds, while the ^31^P signals of other metabolites did not show significant average enhancements.

### 3.3 T_1_ relaxation times

**FIGURE 5:**
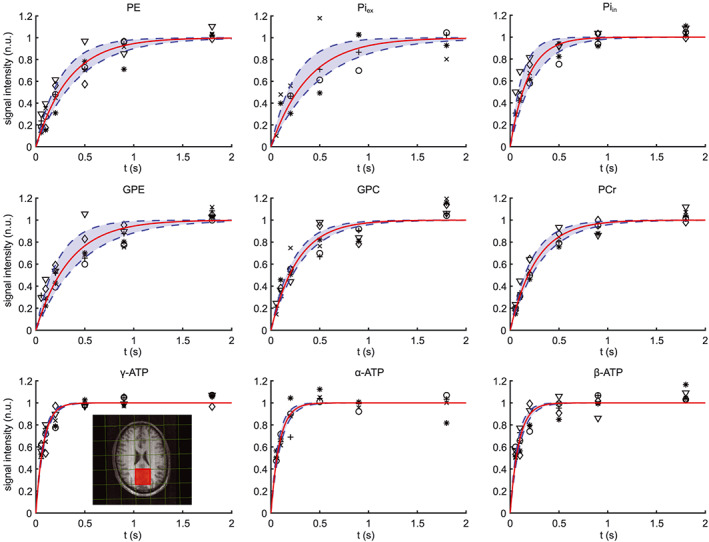
^31^P signal intensity as a function of TR and fit to determine mean T_1_ times of ^31^P spins of nine metabolites in the human brain at 3 T. Markers indicate data points from six volunteers, normalized to the fitted magnetization at thermal equilibrium (M_0_). Average fit of signal curves are displayed in red, ± the standard deviation (blue area). The inset shows the voxel for which the T_1_ measurements were performed (red). n.u., normalized units

Knowledge of T_1_ relaxation times of ^31^P spins is useful to understand their biophysical properties and to optimize pulse sequence TRs. However, for the human brain at 3 T there is little ^31^P T_1_ data available. Therefore, we determined T_1_ values for ^31^P spins of phosphorylated compounds observed in ^31^P spectra from an occipital voxel (inset, Figure 5) by saturation recovery experiments in six volunteers. Normalized data from all volunteers and the curves of the corresponding averaged T_1_s are displayed in Figure 5. For PC and NAD(H) the fit of the recovery curves had a poor quality (R^2^ < 0.7), probably because of their low SNR signals at short TRs, which are then difficult to fit properly next to the larger peaks of PE and α‐ATP. Therefore, these T_1_ curves were not evaluated. T_1_ curves of all other metabolites were successfully fitted (for α‐ATP: *n* = 6; and for Pi_ex_: *n* = 4) and resulted in the mean T_1_ relaxation times as presented in Table 3. Interestingly, the T_1_ values for Pi_in_ and Pi_ex_ are significantly different (Table 3).

**TABLE 3:** Overview of T1 relaxation times (in seconds) of ^31^P spin systems of metabolites in the occipito–temporal–parietal (OPT) region of the human brain at field strengths of 1.5–9.4 T. The results of the current study are in bold, which includes the averages of data from 6 or 4(^†^) volunteers. T1 s of Pi_ex_ and Pi_in_ were significantly different (unpaired Student's t‐test: *P* = 0.015). T1 values of PC and NAD(H) were not included because in short‐TR spectra the fitting of these resonances could not be performed reliably. Below 3 T no separate T1 times are reported for PE and PC, Pi_ex_ and Pi_in_, GPE and GPC

Reference	*B*_*0*_, T	n	Method		PE	PC	Pi_ex_	Pi_in_	GPE	GPC	PCr	γ‐ATP	α‐ATP	NAD^+^	β‐ATP
Luyten et al^24^	1.5	ns	IR	ISIS	2.74 (± 1.04)			1.47 (± 0.12)	1.64 (± 0.08)		3.29 (± 0.53)	1.36 (± 0.30)	0.97 (± 0.12)		1.03 (± 0.36)
Roth et al^25^	1.5	ns	IR	ISIS	1.42 (± 0.43)			1.45 (± 0.26)	1.32 (± 0.14)		3.14 (± 0.47)	0.65 (± 0.15)	0.85 (± 0.21)		0.80 (± 0.16)
Hubesch et al^26^	2.0	3	FIR	ISIS	1.70 (± 0.20)			1.40 (± 0.40)	1.30 (± 0.10)		2.70 (± 0.20)	0.60 (± 0.00)	1.00 (± 0.20)		0.70 (± 0.10)
Merboldt et al^27^	2.0	6	PS	STEAM	4.00 (± 20%)			2.50 (± 20%)	2.00 (± 20%)		3.00 (± 20%)	0.70 (± 20%)	0.70 (± 20%)		1.00 (± 20%)
Klomp et al^28^	3.0	5	IR	ISIS	6.67 (± 0.47)	5.27 (± 1.40)			7.75 (± 0.94)	7.03 (± 0.51)					
Peeters et al	3.0	6	PS	SI	3.42 (± 0.93)		3.82 (± 1.40)^†^	1.84 (± 0.65)	3.44 (± 1.21)	2.72 (± 0.45)	2.66 (± 0.52)	0. 80 (± 0.08)	0.88 (± 0.14)^†^		0.89 (± 0.14)
Lu et al^29^	4.0	12	IR	SC							4.36 (± 0.25)				
Chu et al^30^	4.1	5	IR	SI				1.59 (± 0.16)			2.39 (± 0.01)	0.79 (± 0.11)			
Ren et al^31^	7.0	6	IR	SC	6.33 (± 1.10)	4.31 (± 1.04)	5.80 (± 1.07)	3.70 (± 0.46)	6.79 (± 0.95)	5.82 (± 0.88)	3.39 (± 0.17)	1.70 (± 0.15)	1.35 (± 0.14)	2.07 (± 0.13)	1.13 (± 0.09)
Lei et al^32^	7.0	9	IR	SC	4.78 (± 0.99)			3.19 (± 0.49)	4.06 (± 1.21)	4.01 (± 1.28)	3.37 (± 0.29)	1.27 (± 0.22)	1.26 (± 0.07)		1.02 (± 0.12)
Lu et al^29^	7.0	10	IR	SC							3.54 (± 0.21)				
Pohmann et al^33^	9.4	5	IR	CH‐AC	5.13 (± 0.52)	3.02 (± 0.42)		3.24 (± 0.26)	4.17 (± 0.29)	4.27 (± 0.25)	2.50 (± 0.03)	1.46 (± 0.16)	0. 99 (± 0.13)	1.52 (± 0.58)	

Acquisition methods: IR, inversion recovery; FIR, fast inversion recovery; PS, progressive saturation. Volume selection methods: ISIS, image‐selected in vivo spectroscopy; STEAM, stimulated echo acquisition mode; SI, spectroscopic imaging; SC, surface coil; CH‐AC, two channels of an array coil; n indicates the number of included subjects; ns, not specified.

We compared the T_1_ relaxation times of this study to values obtained at field strengths from 1.5 to 9.4 T as reported by others^24^, ^25^, ^26^, ^27^, ^28^, ^29^, ^30^, ^31^, ^32^, ^33^, ^34^ (Table 3) and concluded that all our T_1_ values are within the range that could be expected from these data. For example, for PCr we found an average T_1_ of 2.66 ± 0.52 seconds (n = 6), while reported values range from 3.29 seconds at 1.5 T to 2.55 seconds at 9.4 T (Table 3).

## 4 DISCUSSION

In this work we employed a custom‐built coil setup consisting of a double tuned ^1^H/^31^P birdcage transmit/receive coil and a ^31^P eight‐channel receive‐array insert to demonstrate that, by combining ^1^H decoupling and ^1^H‐^31^P NOE enhancement, high quality whole‐brain ^31^P MRSI data can be obtained in a clinically feasible measurement time of 13 minutes and within SAR limits. With this setup we determined the ^31^P resonance linewidth decrease by ^1^H decoupling, ^1^H‐^31^P NOE enhancement and T_1_ relaxation times of ^31^P spins of metabolites in the OTP region of the healthy human brain at 3 T. Previously, we reported that with this receive array coil, an average SNR gain factor of 1.4 can be obtained for the whole brain, up to a factor of 3.2 in superficial brain areas.^14^ As a result of the improvements in linewidth and SNR we were able to determine, at 3 T with ^31^P MRSI, the redox state of the brain from the NAD+/NADH ratio, and to observe a peak for Pi_ex_.

**FIGURE 6:**
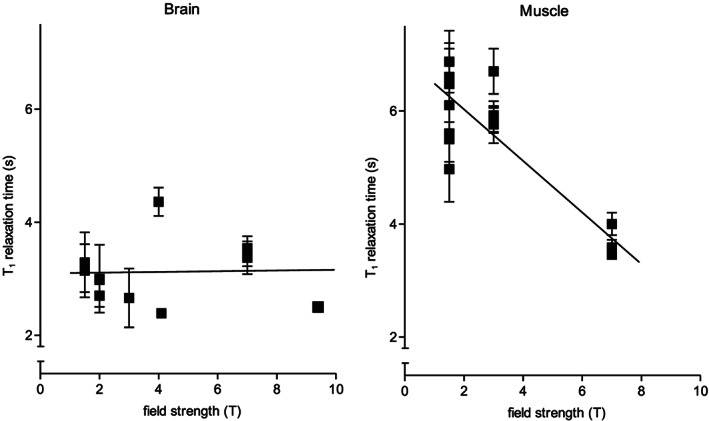
T_1_ relaxation times of PCr as a function of the main magnetic field strength from 1.5 to 9.4 T in the occipito–temporal–parietal (OTP) region of the human brain (left) and from 1.5 to 7 T in the human tibialis anterior and gastrocnemius muscle (right). For convenience, we performed linear regression, which is indicated by solid lines (brain: slope = 0.006 s/T; and muscle: slope = −0.456 s/T, *P* (slope ≠ 0) < 0.001)

As there is little data available on T_1_ relaxation times of ^31^P spin systems of brain metabolites at 3 T,^28^ we determined these times for nearly all detectable metabolites in the ^31^P spectrum. To obtain spectra with good SNR, we performed progressive saturation experiments in a relatively large MRSI voxel containing both white and gray matter. Our T_1_ times are comparable with those in a study performed at 2 T also using progressive saturation.^27^ We compared our T_1_ values with those obtained at different field strengths (Table 3). Some variations in T_1_ values may occur due to the use of different acquisition sequences, number of subjects, and data postprocessing.^34^, ^35^ In addition, different localization procedures were followed including the use of surface coils only.^29^, ^32^ Nevertheless, no specific decrease for the T_1_ values of any of the human brain ^31^P metabolite spins as a function of field strength is observed up to 9.4 T (Figure 6, Table 3). For example, the average T_1_ (± SEM) of PCr at 1.5–2.0 T is 3.0 ± 0.1 seconds, at 3.0–4.1 T it is 3.3 ± 0.5 seconds, and at 7.0–9.4 T it is 3.1 ± 0.3 seconds. And for α‐ATP spins the average T_1_ is 0.9 ± 0.1 seconds at 1.5–2.0 T and 1.1 ± 0.2 seconds at 7.0–9.4 T. A similar observation was made earlier,^36^ but a more recent report on PCr and ATP in human and rat brain suggested a decrease in T_1_ from 4 to 16.4 T.^29^

These observations are in contrast to the reported T_1_ values of ^31^P spins of all phosphorylated metabolites for which resonances are observed in ^31^P MR spectra of skeletal muscles. At 1.5 T these spin systems have longer T_1_ values than the corresponding spin systems in the brain, and they decline significantly at increasing field strengths up to 7 T. For example, the reported T_1_ relaxation time of PCr in human calf muscle or tibialis anterior is 5.0–6.9 seconds at 1.5–3 T and decreases to 3.5–4.0 seconds at 7 T (Figure 6).^24^, ^25^, ^35^, ^37^, ^38^, ^39^, ^40^, ^41^

The T_1_ times of metabolite ^31^P spin systems are considered to be determined by dipolar and chemical shift anisotropy (CSA) relaxation mechanisms.^1^ As CSA relaxation is proportional to the square of the magnetic field strength and dipolar relaxation decreases as a function of the field strength, it is generally assumed that in T_1_ relaxation of ^31^P spins in muscles that CSA becomes dominant from 1.5 to 7 T.^35^ This is clearly not the case in the brain. In general, T_1_ relaxation of ^31^P spins is a complicated function of rotational correlation times, dipolar relaxation rates, CSA relaxation and chemical exchange rates.^42^ If we assume that the rotational correlation time is comparable for all metabolites, then dipolar relaxation is most likely the dominant contribution to the T_1_ times of ^31^P spins in brain. Or a balance exists between decreasing dipolar and increasing CSA relaxation rates between 1.5 and 9.4 T, resulting in virtually unaffected T_1_ values for this range of field strengths.

As T_1_s of polyphosphates (eg, ATP) are much shorter than those of monophosphates (PME, PDE) at 7 T, it was suggested that the mechanism of ^31^P‐^31^P dipolar interactions rather than CSA is important in T_1_ relaxation of brain metabolite ^31^P spins at this field strength.^31^ In addition, trace amounts of divalent paramagnetic ions such as Mn^2+^ can form complexes with ATP, which could also contribute to the shorter T_1_ values of its phosphates.^1^ Because the γ‐phosphate of γ‐ATP is in chemical exchange with those of PCr and Pi_in_, the T_1_ relaxation times of each of these ^31^P components will be the fractional weighted average of the T_1_ values and relative tissue levels of the exchanging partners, which is different between brain and muscle.

Because there is also limited quantitative steady‐state ^1^H‐^31^P NOE enhancement data available for ^31^P spins in the brain at 3 T, we also determined these. The steady‐state NOE enhancement was largest for PME and PDE (up to ~ 50%), whereas the effect on ATP was the smallest (~ 15%). Interestingly, NOE values at 3 T were comparable with those at 7 T, ie, for most evaluated metabolites we found similar to slightly higher enhancements.^21^, ^32^ Moreover, NOE enhancement of PCr in our study is comparable with the measured enhancement at 1.5 T (~ 30%).^23^ As these NOEs depend on dipolar relaxation, this would imply that T_1_ values of ^31^P metabolites essentially remain the same between 1.5 and 7 T, as was concluded above. In muscles, the ^1^H‐^31^P NOE enhancement of PCr decreases from 64–75% at 1.5–3 T to 35% at 7 T,^38^, ^43^ indicating that in muscles CSA is important. In confirmation we observed a decrease in ^31^P‐^31^P NOE of ATP phosphates in skeletal muscles from 3 to 7 T (unpublished results). All these observations strongly indicate that CSA plays a dominant role in T_1_ relaxation of ^31^P metabolite spins in muscle but not in the brain with increasing field strength.

NOE enhancement can increase the reproducibility of measuring ^31^P resonances,^21^ but, as for T_1_ relaxation times, has to be taken into account in the quantification of metabolite ratios or tissue levels, in particular if NOEs are expected to be affected by changes in tissue condition.

By means of ^1^H decoupling the linewidth of resonances of proton‐coupled phosphates measured at 3 T decreased, roughly in agreement with the value of the three‐bond ^1^H‐^31^P J‐coupling of the compounds involved.^1^ This decoupling also increased the SNR of the compound resonances. A higher main magnetic field is commonly expected to improve spectral resolution and SNR. The field strength dependence of spectral resolution in ^31^P MRS of the brain has been studied in detail by Lu et al.^29^ The linewidth we find for PCr at 3 T (~ 0.14 ppm, 7.3 Hz) fits to the field strength dependency presented in that study, in which the PCr linewidth in ppm is shown to decrease to ~ 0.1 ppm in the occipital brain region at 7 T. For the two resolved ^31^P‐^31^P coupled resonances of α‐ATP, we find for each peak a linewidth of ~ 17 Hz at 3 T. At field strengths of 4 T or higher these are not resolved anymore because the absolute linewidths exceed the ^31^P‐^31^P J‐coupling constant of 16–17 Hz.^44^ If we take a coupling constant of 16 Hz into account, the total linewidth of the α‐ATP peak is ~ 33 Hz, which is in agreement with the data reported in Lu et al.^29^ Even although linewidths in ppm, and thus spectral resolution, improve somewhat at 7 T and higher field strengths compared with 3 T,^29^ all resonances identified in spectra at 7 T can also be separately assessed at 3 T using ^1^H decoupling. For example, resonances for PE, PC and GPE and GPC are well resolved, and we also show that at sufficient SNR an additional phosphate peak can be detected at ~ 5.3 ppm. This Pi_ex_ is assumed to arise from an extracellular compartment (eg, blood) and may be employed to assess brain diseases.^20^, ^45^ We observed that the T_1_ of Pi_in_ is shorter than that of Pi_ex_, which has been attributed to the involvement of Pi_in_ in chemical exchange with the γ‐ATP phosphate, which is subjected to efficient dipolar relaxation.^31^

It has been demonstrated that NADH and NAD^+^ can be separately quantified at 4 T using ^1^H decoupling, with the suggestion that this should also be possible at 3 T.^2^ Indeed, we observed that the two ^31^P doublets of NAD^+^ can be analyzed separately from the ^31^P peak of NADH at 3 T when employing ^1^H decoupling and NOE, even for MRSI with voxel volumes that are several times smaller than the detection volume employed in the study at 4 T. With the assumptions of equal linewidths and fixed chemical shift difference between NAD^+^ and NADH, our calculated NAD^+^ and NADH tissue concentrations and redox ratios are comparable with those observed at 4 and 7 T.^2^, ^31^ These concentrations, and that of Pi_ex_, can be determined more accurately by measuring larger voxels for better SNR, as is commonly done in studies at higher fields.^2^, ^20^ NAD(H) quantification could be further refined by including resonances of uridine diphosphate glucose (UDPG), of which one phosphate co‐resonates with those of the NAD(H) phosphates.^46^, ^47^ However, larger voxels and/or longer measurement times than employed in the current 3 T study are needed for proper detection of the UDPG signals.

A potential problem for quantitation of the GPC and GPE resonance at 3 T is a broad signal from immobile phosphates centered at ~ 2.25 ppm. This signal broadens at higher fields to become part of the spectral baseline, but its visibility may be of interest in certain conditions.^48^ As its linewidth is much larger than that of the other ^31^P resonances, it is easy to separate it from these resonances by either fitting or filtering.

The increased intrinsic SNR of fields higher than 3 T is obviously an advantage, but the assumed additional benefit of shorter T_1_s and thus faster repetition times does not seem to count for ^31^P MRS of the brain, at least up to 7 T. Additionally, NOE effects at 3 T are comparable with those at 7 T.^21^, ^32^ Furthermore, inhomogeneous ^1^H transmit fields may complicate the application of ^1^H‐^31^P NOE and ^1^H decoupling techniques for the whole brain at higher fields, and SAR limitations may require longer repetition times for additional ^1^H irradiation. Therefore, we conclude that when the best possible spatial resolution is not a premium requirement for localized ^31^P MRS, its performance at 3 T using a phased array receive with ^1^H decoupling and NOE is an excellent and more accessible alternative to ^31^P MRS at 7 T and higher. Further improvements of ^31^P MRSI are expected to be possible, eg, by speeding up data acquisition using compressed sensing methods, and by advanced data reconstructions.^49^, ^50^

## References

[1] de Graaf RA . In Vivo NMR Spectroscopy: Principles and Techniques. Third ed. Chichester, England: John Wiley & Sons Ltd; 2018.

[2] Lu M , Zhu X‐H , Chen W . In vivo 31 P MRS assessment of intracellular NAD metabolites and NAD^+^/NADH redox state in human brain at 4 T. NMR Biomed. 2016;29:1010‐1017. 27257783 10.1002/nbm.3559 PMC4909585

[3] Hattingen E , Bähr O , Rieger J , Blasel S , Steinbach J , Pilatus U . Phospholipid metabolites in recurrent glioblastoma: in vivo markers detect different tumor phenotypes before and under antiangiogenic therapy. PLoS ONE. 2013;8:e56439. 23520454 10.1371/journal.pone.0056439 PMC3592858

[4] Pettegrew JW , Panchalingam K , Klunk WE , McClure RJ , Muenz LR . Alterations of cerebral metabolism in probable Alzheimer's disease: a preliminary study. Neurobiol Aging. 1994;15:117‐132. 8159258 10.1016/0197-4580(94)90152-x

[5] Rango M , Bonifati C , Bresolin N . Parkinson's disease and brain mitochondrial dysfunction: a functional phosphorus magnetic resonance spectroscopy study. J Cereb Blood Flow Metab. 2006;26:283‐290. 16094320 10.1038/sj.jcbfm.9600192

[6] Grond J , Gerson JR , Laxer KD , Hugg JW , Matson GB , Weiner MW . Regional distribution of interictal 31P metabolic changes in patients with temporal lobe epilepsy. Epilepsia. 1998;39:527‐536. 9596206 10.1111/j.1528-1157.1998.tb01416.x PMC2735262

[7] Weber‐Fahr W , Englisch S , Esser A , et al. Altered phospholipid metabolism in schizophrenia: a phosphorus 31 nuclear magnetic resonance spectroscopy study. Psychiatry Res Neuroimaging. 2013;214:365‐373. 10.1016/j.pscychresns.2013.06.011 24045051

[8] Rijpma A , van der Graaf M , Meulenbroek O , Olde Rikkert MGM , Heerschap A . Altered brain high‐energy phosphate metabolism in mild Alzheimer's disease: a 3‐dimensional 31P MR spectroscopic imaging study. NeuroImage Clin. 2018;18:254‐261. 29876246 10.1016/j.nicl.2018.01.031 PMC5987799

[9] Oz G , Alger JR , Barker PB , et al. Clinical proton MR spectroscopy in central nervous system disorders. Radiology. 2014;270:658‐679. 24568703 10.1148/radiol.13130531 PMC4263653

[10] van de Bank BL , Orzada S , Smits F , et al. Optimized ^31^P MRS in the human brain at 7 T with a dedicated RF coil setup. NMR Biomed. 2015;28:1570‐1578. 26492089 10.1002/nbm.3422 PMC4744789

[11] Greenman RL , Rakow‐Penner R . Evaluation of the RF field uniformity of a double‐tuned 31P/1H birdcage RF coil for spin‐echo MRI/MRS of the diabetic foot. J Magn Reson Imaging. 2005;22:427‐432. 16104007 10.1002/jmri.20372

[12] Matson GB , Vermathen P , Hill TC . A practical double‐tuned 1H/31P quadrature birdcage headcoil optimized for 31P operation. Magn Reson Med. 1999;42:173‐182. 10398964 10.1002/(sici)1522-2594(199907)42:1<173::aid-mrm23>3.0.co;2-o

[13] Avdievich NI , Hetherington HP . 4 T Actively detuneable double‐tuned 1H/31P head volume coil and four‐channel 31P phased array for human brain spectroscopy. J Magn Reson. 2007;186:341‐346. 17379554 10.1016/j.jmr.2007.03.001 PMC2677064

[14] van Uden MJ , Peeters TH , Rijpma A , Rodgers CT , Heerschap A , Scheenen TWJ . An 8‐channel receive array for improved 31 P MRSI of the whole brain at 3 T. Magn Reson Med. 2019;82:825‐832. 30900352 10.1002/mrm.27736 PMC6520216

[15] Luyten PR , Bruntink G , Sloff FM , et al. Broadband proton decoupling in human 31P NMR spectroscopy. NMR Biomed. 1989;1:177‐183. 2641284 10.1002/nbm.1940010405

[16] Bachert‐Baumann P , Ermark F , Zabel HJ , Sauter R , Semmler W , Lorenz WJ . In vivo nuclear Overhauser effect in 31P‐(1H) double‐resonance experiments in a 1.5‐T whole‐body MR system. Magn Reson Med. 1990;15:165‐172. 2165209 10.1002/mrm.1910150119

[17] Pohmann R , Von Kienlin M . Accurate phosphorus metabolite images of the human heart by 3D acquisition‐weighted CSI. Magn Reson Med. 2001;45:817‐826. 11323808 10.1002/mrm.1110

[18] Brown MA . Time‐domain combination of MR spectroscopy data acquired using phased‐array coils. Magn Reson Med. 2004;52:1207‐1213. 15508170 10.1002/mrm.20244

[19] Lu M , Zhu XH , Zhang Y , Chen W . Intracellular redox state revealed by in vivo 31P MRS measurement of NAD^+^ and NADH contents in brains. Magn Reson Med. 2014;71:1959‐1972. 23843330 10.1002/mrm.24859 PMC4384818

[20] Ren J , Shang T , Sherry AD , Malloy CR . Unveiling a hidden 31P signal coresonating with extracellular inorganic phosphate by outer‐volume‐suppression and localized 31P MRS in the human brain at 7 T. Magn Reson Med. 2018;80:1289‐1297. 29427295 10.1002/mrm.27121 PMC6085175

[21] Lagemaat MW , van de Bank BL , Sati P , Li S , Maas MC , Scheenen TW . Repeatability of ^31^P MRSI in the human brain at 7 T with and without the nuclear Overhauser effect. NMR Biomed. 2016;29:256‐263. 26647020 10.1002/nbm.3455 PMC4769102

[22] Du F , Zhu XH , Qiao H , Zhang X , Chen W . Efficient in vivo 31P magnetization transfer approach for noninvasively determining multiple kinetic parameters and metabolic fluxes of ATP metabolism in the human brain. Magn Reson Med. 2007;57:103‐114. 17191226 10.1002/mrm.21107

[23] Murphy‐Boesch J , Stoyanova R , Srinivasan R , et al. Proton‐decoupled 31P chemical shift imaging of the human brain in normal volunteers. NMR Biomed. 1993;6:173‐180. 8394101 10.1002/nbm.1940060302

[24] Luyten PR , Groen JP , Vermeulen JWAH , den Hollander JA . Experimental approaches to image localized human 31P NMR spectroscopy. Magn Reson Med. 1989;11:1‐21. 2747510 10.1002/mrm.1910110102

[25] Roth K , Hubesch B , Meyerhoff DJ , et al. Noninvasive quantitation of phosphorus metabolites in human tissue by NMR spectroscopy. J Magn Reson. 1989;81:299‐311.

[26] Hubesch B , Sappey‐Marinier D , Roth K , Meyerhoff DJ , Matson GB , Weiner MW . P‐31 MR spectroscopy of normal human brain and brain tumors. Radiology. 1990;174:401‐409. 2296651 10.1148/radiology.174.2.2296651

[27] Merboldt KD , Chien D , Hanicke W , Gyngell ML , Bruhn H , Frahm J . Localized P‐31 NMR‐spectroscopy of the adult human brain in vivo using stimulated‐echo (steam) sequences. J Magn Reson. 1990;89:343‐361.

[28] Klomp DWJ , Wijnen JP , Scheenen TWJ , Heerschap A . Efficient 1H to 31P polarization transfer on a clinical 3 T MR system. Magn Reson Med. 2008;60:1298‐1305. 19030163 10.1002/mrm.21733

[29] Lu M , Chen W , Zhu XH . Field dependence study of in vivo brain 31P MRS up to 16.4 T. NMR Biomed. 2014;27:1135‐1141. 25070004 10.1002/nbm.3167 PMC4180101

[30] Chu WJ , Mason GF , Hetherington HP . Phosphorus metabolic differences in gray and white matter: 31P NMR spectroscopic imaging studies of human brain at 4 T. In: *Proceedings of the 5th Annual Meeting of ISMRM,*. Vancouver, Canada; 1997.

[31] Ren J , Sherry AD , Malloy CR . 31 P‐MRS of healthy human brain: ATP synthesis, metabolite concentrations, pH, and T 1 relaxation times. NMR Biomed. 2015;28:1455‐1462. 26404723 10.1002/nbm.3384 PMC4772768

[32] Lei H , Zhu X‐H , Zhang X‐L , Ugurbil K , Chen W . In vivo 31P magnetic resonance spectroscopy of human brain at 7 T: an initial experience. Magn Reson Med. 2003;49:199‐205. 12541238 10.1002/mrm.10379

[33] Pohmann R , Raju S , Scheffler K. T1 values of phosphorus metabolites in the human visual cortex at 9.4 T. In: *Proc Intern Soc Magn Reson Med*. Paris; 2018:3394.

[34] Bojorquez JZ , Bricq S , Acquitter C , Brunotte F , Walker PM , Lalande A . What are normal relaxation times of tissues at 3 T? Magn Reson Imaging. 2017;35:69‐80. 27594531 10.1016/j.mri.2016.08.021

[35] Bogner W , Chmelik M , Schmid AI , Moser E , Trattnig S , Gruber S . Assessment of 31 P relaxation times in the human calf muscle: a comparison between 3 T and 7 T in vivo. Magn Reson Med. 2009;62:574‐582. 19526487 10.1002/mrm.22057

[36] Qiao H , Zhang X , Zhu XH , Du F , Chen W . In vivo 31P MRS of human brain at high/ultrahigh fields: a quantitative comparison of NMR detection sensitivity and spectral resolution between 4 T and 7 T. Magn Reson Imaging. 2006;24:1281‐1286. 17145398 10.1016/j.mri.2006.08.002 PMC2610491

[37] Buchthal SD , Thoma WJ , Taylor JS , Nelson SJ , Brown TR . In vivo1 values of phosphorus metabolites in human liver and muscle determined at 1.5 T by chemical shift imaging. NMR Biomed. 1989;2:298‐304. 2641903 10.1002/nbm.1940020520

[38] Brown TR , Stoyanova R , Greenberg T , Srinivasan R , Murphy‐Boesch J . NOE enhancements and T1 relaxation times of phosphorylated metabolites in human calf muscle at 1.5 Tesla. Magn Reson Med. 1995;33:417‐421. 7760710 10.1002/mrm.1910330316

[39] Thomsen C , Jensen KE , Henriksen O . In vivo measurements of T1 relaxation times of 31P‐metabolites in human skeletal muscle. Magn Reson Imaging. 1989;7:231‐234. 2716489 10.1016/0730-725x(89)90709-1

[40] Newcomer BR , Boska MD . T1 measurements of 31P metabolites in resting and exercising human gastrocnemius/soleus muscle at 1.5 Tesla. Magn Reson Med. 1999;41:486‐494. 10204871 10.1002/(sici)1522-2594(199903)41:3<486::aid-mrm10>3.0.co;2-#

[41] Parasoglou P , Xia D , Chang G , Regatte RR . 3D‐mapping of phosphocreatine concentration in the human calf muscle at 7 T: comparison to 3 T. Magn Reson Med. 2013;70:1619‐1625. 23390003 10.1002/mrm.24616 PMC3657590

[42] Nabuurs C , Huijbregts B , Wieringa B , Hilbers CW , Heerschap A . 31P saturation transfer spectroscopy predicts differential intracellular macromolecular association of ATP and ADP in skeletal muscle. J Biol Chem. 2010;285:39588‐39596. 20884612 10.1074/jbc.M110.164665 PMC3000940

[43] Rink K , Berger MC , Korzowski A , et al. Nuclear‐Overhauser‐enhanced MR imaging of 31P‐containing metabolites: multipoint‐Dixon vs. frequency‐selective excitation. Magn Reson Imaging. 2015;33:1281‐1289. 26248272 10.1016/j.mri.2015.07.017

[44] Jung WI , Staubert A , Widmaier S , et al. Phosphorus J‐coupling constants of ATP in human brain. Magn Reson Med. 1997;37:802‐804. 9126956 10.1002/mrm.1910370525

[45] Novak J , Wilson M , MacPherson L , Arvanitis TN , Davies NP , Peet AC . Clinical protocols for 31P MRS of the brain and their use in evaluating optic pathway gliomas in children. Eur J Radiol. 2014;83:e106‐e112. 24331847 10.1016/j.ejrad.2013.11.009 PMC4029084

[46] de Graaf RA , De Feyter HM , Brown PB , Nixon TW , Rothman DL , Behar KL . Detection of cerebral NAD^+^ in humans at 7 T. Magn Reson Med. 2017;78:828‐835. 27670385 10.1002/mrm.26465 PMC5366282

[47] Xin L , Ipek Ö , Beaumont M , et al. Nutritional ketosis increases NAD^+^/NADH ratio in healthy human brain: an in vivo study by 31P‐MRS. Front Nutr. 2018;5:1‐8. 30050907 10.3389/fnut.2018.00062 PMC6052097

[48] van der Kemp WJ , Stehouwer BL , Runge JH , et al. Glycerophosphocholine and glycerophosphoethanolamine are not the main sources of the in vivo ^31^P MRS phosphodiester signals from healthy fibroglandular breast tissue at 7 T. Front Oncol. 2016;6:29. 26913240 10.3389/fonc.2016.00029 PMC4753293

[49] Nassirpour S , Chang P , Kirchner T , Henning A . Over‐discretized SENSE reconstruction and B 0 correction for accelerated non‐lipid‐suppressed 1 H FID MRSI of the human brain at 9.4 T. NMR Biomed. 2018;31:e4014. 30334288 10.1002/nbm.4014

[50] Santos‐Díaz A , Noseworthy MD . Comparison of compressed sensing reconstruction algorithms for 31P magnetic resonance spectroscopic imaging. Magn Reson Imaging. 2019;59:88‐96. 30853562 10.1016/j.mri.2019.03.006

